# Supplementary material for: Adaptation Strategies of Seedling Root Response to Nitrogen and Phosphorus Addition
Source: Plants (Basel). 2024 Feb 15;13(4):536. doi: 10.3390/plants13040536 (PMC10892864; doi:10.3390/plants13040536)
Supplement: Supplementary file 1 [file plants-13-00536-s001.zip › plants-2850747-supplementary.pdf]

Supplementary materials

Table S1. Soil physical and chemical properties under different nitrogen and phosphorus addition treatments

Treatment	pH	TC mg/g	TN mg/g	AN mg/g	NN mg/g	TP mg/g	AP mg/g
N_0P_0	4.99±0.0200	2.463±0.0189	0.408±0.0696	0.01±0.0005	0.0009±0.00011	0.033±0.0017	0.044±0.0022
N_0P_1	4.79±0.0115	1.906±0.0049	0.309±0.0349	0.009±0.0005	0.0027±0.00024	0.017±0.007	0.044±0.0012
N_0P_2	5.19±0.0586	2.431±0.0051	0.418±0.0282	0.008±0.0003	0.0008±0.00008	0.319±0.2486	0.048±0.0034
N_0P_3	5.230±0.0173	2.268±0.0134	0.322±0.0438	0.009±0.0005	0.0001±0.00004	0.046±0.0057	0.056±0.0056
N_1P_0	4.817±0.0120	2.04±0.0118	0.345±0.0204	0.007±0.0002	0.0044±0.00003	0.224±0.1778	0.053±0.0047
N_1P_1	4.747±0.0233	2.437±0.0540	0.473±0.0179	0.008±0.0001	0.0043±0.00317	0.048±0.0025	0.043±0.0009
N_1P_2	4.773±0.012	2.175±0.3726	0.315±0.0609	0.007±0.0002	0.0005±0.00003	0.019±0.0083	0.054±0.0046
N_1P_3	4.917±0.0088	2.091±0.1925	0.314±0.051	0.007±0.0003	0.0003±0.00002	0.05±0.0083	0.067±0.0111
N_2P_0	4.167±0.0033	2.144±0.0415	0.35±0.0452	0.01±0.0004	0.0281±0.00041	0.039±0.0073	0.044±0.0009
N_2P_1	4.073±0.0033	1.99±0.13470	0.382±0.0692	0.023±0.0002	0.0182±0.00015	0.011±0.0063	0.041±0.0037
N_2P_2	4.117±0.0033	1.695±0.0744	0.319±0.0198	0.008±0.0002	0.0096±0.00006	0.012±0.0055	0.048±0.0020
N_2P_3	4.047±0.0033	1.585±0.0389	0.307±0.0324	0.013±0.0002	0.0129±0.0001	0.029±0.0094	0.047±0.0011
N_3P_0	3.950±0.0058	2.056±0.2687	0.442±0.0965	0.061±0.0011	0.0215±0.00015	0.039±0.0094	0.051±0.0067
N_3P_1	3.957±0.0033	1.996±0.0126	0.436±0.0554	0.028±0.0004	0.0314±0.00016	0.061±0.0444	0.065±0.0066
N_3P_2	3.917±0.0203	2.008±0.0064	0.47±0.012	0.052±0.0002	0.0297±0.00014	0.023±0.0102	0.049±0.0013
N_3P_3	3.960±0.0100	2.279±0.0028	0.408±0.0457	0.079±0.0007	0.0287±0.00026	0.081±0.0125	0.046±0.0001

Note： TC：Total carbon；TN：Total nitrogen；AN：Ammonium nitrogen；NN：Nitrate nitrogen；TP：Total phosphorus；AP：Available phosphorus.

Table S2. Level of nitrogen and phosphorus addition

Treatment	Nitrogen addition concentration （g m^{-2} a^{-1}）	NH_4Cl （g/ per pot）	Phosphorus addition concentration （g m^{-2} a^{-1}）	NaH_2PO_4 （g/ per pot）
N_0P_0	0	0	0	0
N_0P_1	0	0	0.6	0.070
N_0P_2	0	0	2.4	0.280
N_0P_3	0	0	4.2	0.490
N_1P_0	3.6	0.414	0	0
N_1P_1	3.6	0.414	0.6	0.070
N_1P_2	3.6	0.414	2.4	0.280
N_1P_3	3.6	0.414	4.2	0.490
N_2P_0	14.4	1.657	0	0
N_2P_1	14.4	1.657	0.6	0.070
N_2P_2	14.4	1.657	2.4	0.280
N_2P_3	14.4	1.657	4.2	0.490
N_3P_0	25.2	2.900	0	0
N_3P_1	25.2	2.900	0.6	0.070
N_3P_2	25.2	2.900	2.4	0.280
N_3P_3	25.2	2.900	4.2	0.490

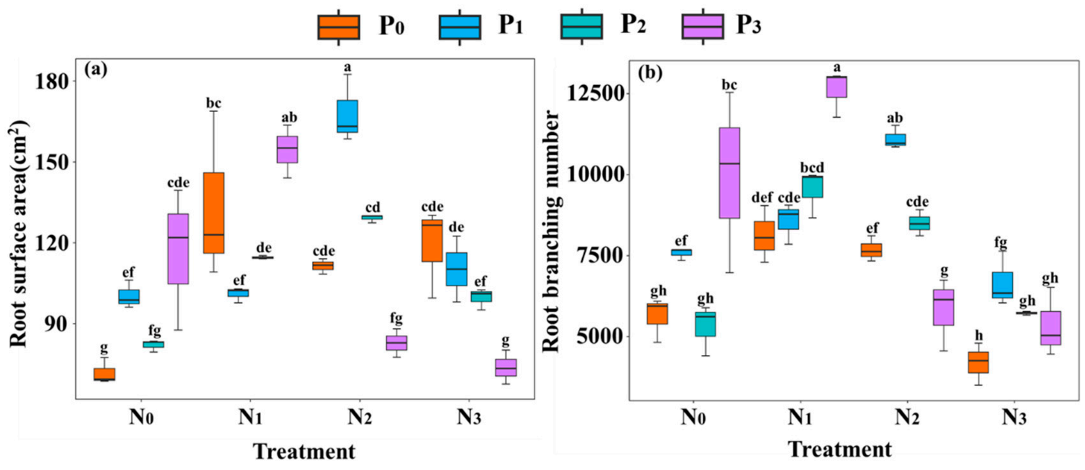

Figure S1. Effect of nitrogen and phosphorus addition on root traits of *Castanopsis kawakamii* seedlings

Notes: (a), (b) represent root branching number, root surface area, respectively. N_0P_i: single phosphorus addition treatment, N_iP_0: single nitrogen addition treatment, N_1P_i: low nitrogen and phosphorus interaction treatment, N_2P_i: medium nitrogen and phosphorus interaction treatment, N_3P_i: high nitrogen and phosphorus interaction treatment, where i=1,2,3. The differences represented by different lowercase letters between treatments with different nitrogen and phosphorus concentrations added are significant ($p<0.05$). The same applies to the following.

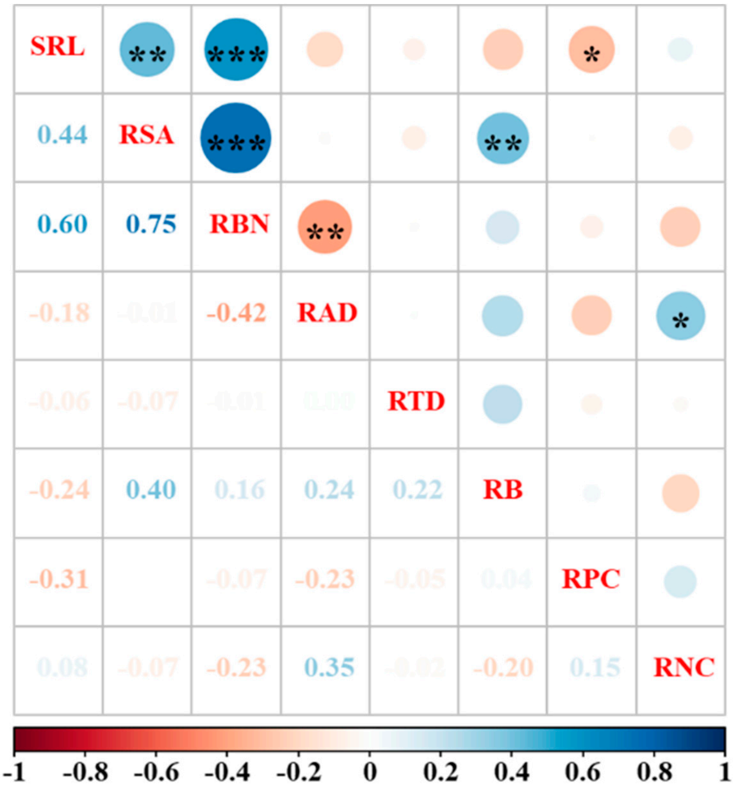

Figure S2. Correlation between root traits of *Castanopsis kawakamii* seedlings

Note: In the figure, SRL, RSA, RBN, RAD, RTD, RB, RPC, and RNC represent specific root length, root surface area, root branching number, root average diameter, root tissue density, root biomass, phosphorus content, and nitrogen content of the roots, respectively. The same applies to the following. In the figure, * denotes a significant correlation at the 0.05 level, ** denotes a highly significant correlation at the 0.01 level, and *** denotes a highly significant correlation at the 0.001 level.

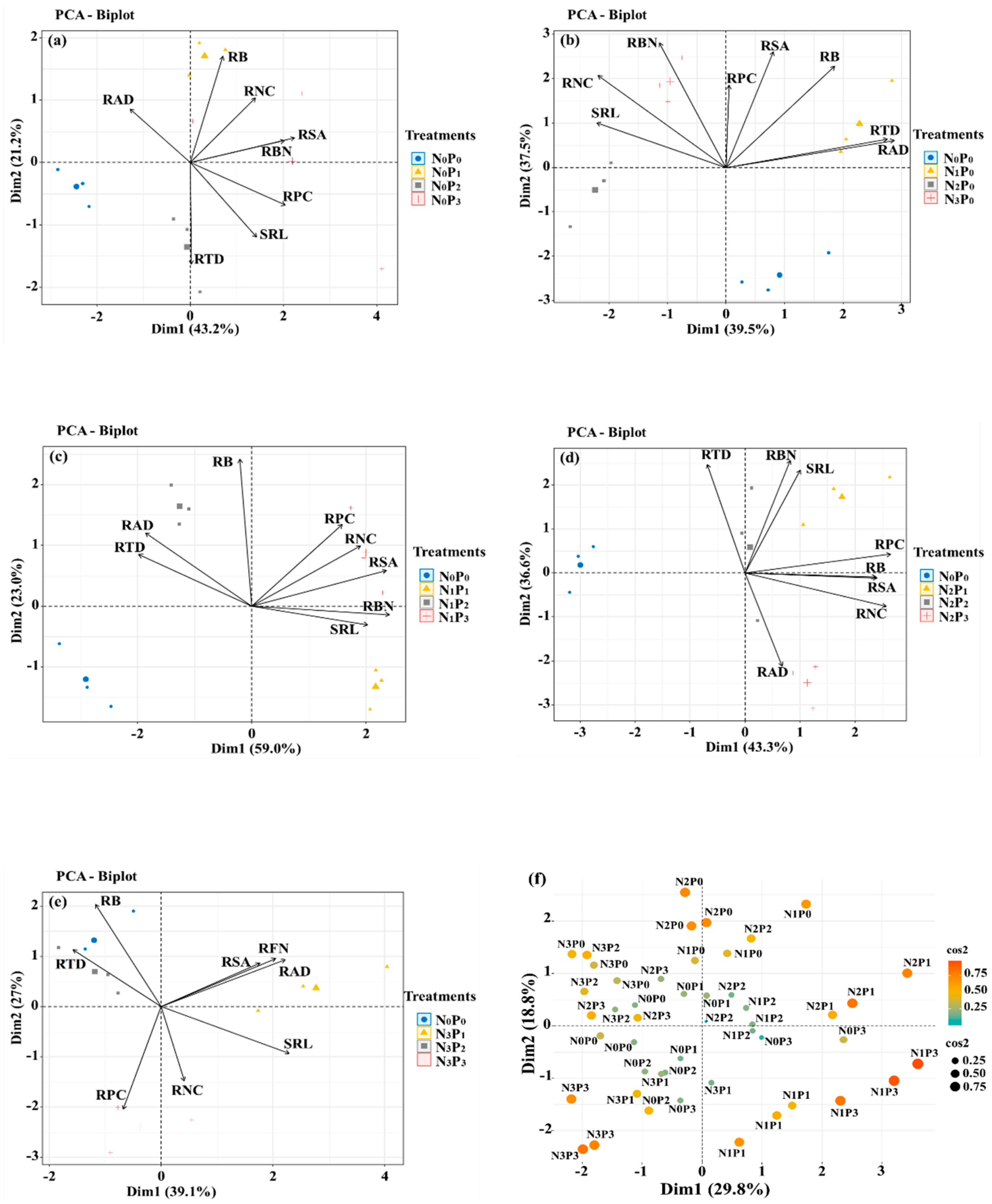

Figure S3. Principal component analysis (PCA) of root traits under different nitrogen and phosphorus addition treatments.

Note: N_0P_0 is a non-nitrogen phosphorus addition treatment, (**a**): Single phosphorus addition treatment (N_0P_i); (**b**): Single nitrogen addition treatment (N_iP_0); (**c**): Low nitrogen and phosphorus interactive addition treatment (N_1P_i); (**d**): Interactive addition of medium nitrogen and phosphorus(N_2P_i); (**e**): High nitrogen and phosphorus interactive addition treatment (N_3P_i), where, i=1, 2, 3; (f): Scatter contribution plot of

different nitrogen and phosphorus addition treatments. The cos2 value represents the contribution of the variable to the principal component.
